# Integrative Treatment for Tinnitus Combining Repeated Facial and Auriculotemporal Nerve Blocks With Stimulation of Auditory and Non-auditory Nerves

**DOI:** 10.3389/fnins.2022.758575

**Published:** 2022-02-28

**Authors:** Soo Ji Sirh, So Woon Sirh, Hah Yong Mun, Heon Man Sirh

**Affiliations:** ^1^Department of Neurosurgery, Sirh’s Private Pain Clinic, Seoul, South Korea; ^2^Department of Anesthesiology and Pain Medicine, Wiltse Memorial Hospital, Suwon-si, South Korea; ^3^Department of Neurosurgery, Yangju Armed Forces Hospital, Yangju-si, South Korea; ^4^Department of Anesthesiology and Pain Medicine, Sirh’s Private Pain Clinic, Seoul, South Korea

**Keywords:** tinnitus, neuromodulation, auditory nervous system, non-auditory nervous system, trigeminal nerve, facial nerve, vestibulocochlear nerve

## Abstract

**Background:**

Tinnitus is a prevalent condition (>10% of the population) affecting the quality of life of 0.5–3% of the population. Although several treatments have been proposed, most of these lack evidence of efficacy in the treatment of chronic tinnitus. Thus, we aimed to evaluate an integrative treatment strategy for subacute and chronic tinnitus.

**Methods:**

This retrospective chart review study included 55 patients with tinnitus (subacute, *n* = 15; chronic, *n* = 40) who underwent repeated nerve blocks after stimulation of the trigeminal (V) and facial (VII) nerves to modulate the auditory and non-auditory nervous systems via the vestibulocochlear (VIII) cranial nerve pathways. We used a simplified smiley tinnitus-visual analog scale (T-VAS) with scores ranging from 0 to 10 combining the effect of tinnitus loudness, distress, and quality of life as the outcome measure to evaluate the efficacy of our treatment method. Statistical analyses were performed using SPSS (version 18.0, SPSS Inc., Chicago, IL, United States), one-way and two-way analysis of variance.

**Results:**

In more than 87.5% of patients (14/15 subacute, 35/40 chronic), tinnitus disappeared or had significantly reduced by the end of the treatment. The mean T-VAS score reduced significantly from 7.13 to 0.60 in the subacute group and from 7.73 to 1.53 in the chronic group by the end of treatment (*p* < 0.05). The benefits were maintained after treatment cessation and at the 1-year follow-up. The average number of treatment procedures was 9.8 ± 3.589 (range, 5–15) in the subacute group and 9.775 ± 3.717 (range, 5–18) in the chronic group.

**Conclusion:**

Our results show that the proposed integrative approach is highly effective in treating subacute and chronic tinnitus and represents a promising therapeutic approach.

## Introduction

Tinnitus is a phantom auditory sensation perceived as diverse sounds, such as ringing, buzzing, roaring, and crickets, without an external source, and affects all age groups (>10%, range 11.9–30.3%) and quality of life (>3%, range 3.0–30.9%; McCormack et al., 2016). Tinnitus lacks a definitive medical cure (Shore et al., 2016). Additionally, tinnitus is commonly accompanied by hearing loss (Shore et al., 2016).

Tinnitus is a refractory symptom with complex, multifactorial causes, which are mainly sensorineural, somatosensory (a subtype of subjective tinnitus), infectious, drug-related, neurovascular, and idiopathic (Lockwood et al., 2002; Cianfrone et al., 2015). Acute and chronic tinnitus are distinguished by duration, though definitive duration criteria have not been established. To treat chronic tinnitus more effectively, it can be further classified according to diverse criteria, such as etiologies, comorbidities, symptom traits, and psychological impact (Haider et al., 2018).

Recently, Cianfrone et al. (2015) proposed the Tinnitus Holistic Simplified Classification, which considered the diverse underlying mechanisms of tinnitus: (1) auditory tinnitus, (2) somatosensory tinnitus related to musculoskeletal and/or trigeminal disease, (3) psychopathology-related tinnitus, and (4) combined tinnitus with two or all of these three mechanisms.

Recent neuroimaging studies have reported that tinnitus perception is associated with hyperactivity in the auditory cortex (Maudoux et al., 2012) and altered functional connectivity in the brain due to peripheral deafferentation in chronic tinnitus. The affected areas are in the auditory and non-auditory cortices involving the limbic (fronto-insular and parahippocampal gyrus) and visual cortices (Burton et al., 2012; Maudoux et al., 2012), the parahippocampal gyrus, and Heschl’s gyrus (Han et al., 2020).

Although many treatment modalities for tinnitus have been developed, treatment outcomes are unpredictable, varied, and less than satisfactory, possibly because all treatment methods, including neuromodulation and neurostimulation, are usually single or bi-modal rather than integrative treatments designed to simultaneously address the multiple causes and underlying mechanisms of tinnitus (Lockwood et al., 2002; Haider et al., 2018; Mcferran et al., 2019).

Presently, it is relatively less difficult to treat somatosensory tinnitus wherein the causes stem from myofascial, ligament, tendon, and joint problems of the neck (especially the upper cervical spine above C3), temporomandibular joint (TMJ) areas, middle ear muscles, and nerve problems associated with auditory maladaptive neuroplasticity. Some of the treatment interventions include a combination of nerve block (for muscle spasm and auditory nerve dysfunction), trigger-point injection (TPI) (for muscle contracture), and prolotherapy (for ligament and tendon injury, instability of the cervical spine, and TMJ problems).

However, to date, it has been difficult to treat chronic subjective tinnitus accompanied by long-term maladaptive plasticity in the peripheral and central auditory and non-auditory nervous systems, or tinnitus due to psychopathology arising from various causes and underlying neural mechanisms that are poorly understood, especially in humans (Lockwood et al., 2002; Marks et al., 2018; Cederroth et al., 2019; Mcferran et al., 2019).

As tinnitus is mainly a heterogeneous condition (Langguth et al., 2013; Cederroth et al., 2019; Schoisswohl et al., 2019), the tinnitus treatment guidelines do not recommend nerve blocks, electrical stimulation, or acupuncture with lifting-thrusting, twirling-twisting, and reinforcing-reducing manipulations due to the uncertainty regarding its effects. Therefore, an individual-specific intervention combining several concurrent treatment options is needed for optimal outcomes (Haider et al., 2017). However, no evidence-based, curative, integrative treatment approach has been available to address the causes and mechanisms of chronic tinnitus.

**FIGURE 1:**
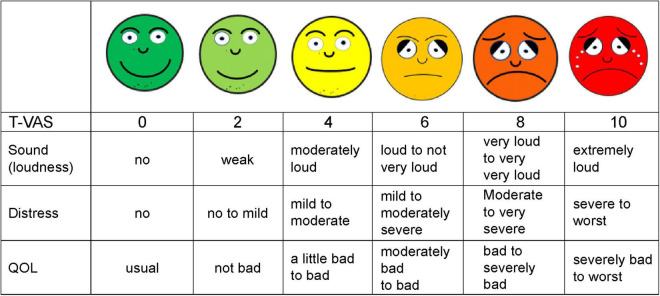
Shown is a simplified smiley tinnitus-visual analog scale (T-VAS) measuring loudness, distress, and quality of life (QOL). 0, no sound, no distress or discomfort, usual QOL; 2, weak, no to mild distress and discomfort, not bad QOL; 4, moderately loud, mild to moderate distress, slightly bad to bad QOL; 5, loud to not very loud, mild to moderately severe distress, moderately bad to bad QOL; 6, loud to not very loud, mild to moderately severe distress, moderately bad to bad QOL; 8, very loud to loud, moderate to very severe distress, bad to severely impaired QOL; 10, extremely or unbearably loud, severe to worst distress, fear, severely impaired to worst QOL.

Hence, we developed a new integrative treatment method combining repeated modified nerve blocks and auditory and non-auditory nerve stimulation without electrical or manual stimulation, with other treatment modalities, including TPI and prolotherapy. Our new integrative treatment is based on the hypothesis that this approach might have superior safety and efficacy in addressing the diverse causes and mechanisms of chronic tinnitus (Lockwood et al., 2002; Marks et al., 2018; Cederroth et al., 2019; Mcferran et al., 2019). Evaluation of the treatment strategy combining the repeated nerve blocks with auditory and non-auditory nervous system stimulation via the trigeminal (V), facial (VII), and vestibulocochlear (VIII) cranial nerves was performed using the smiley T-VAS (Tinnitus Visual Analog Scale) scores (Figure 1) as the outcome measure for subacute and chronic, intractable tinnitus.

## Materials and Methods

This study was approved by the Institutional Review Board (IRB) of the Wiltse Memorial Hospital Joint Research Ethics Committee in Suwon, South Korea (IRB approval number: 2021-W07). The requirement for written informed consent was waived by the IRB as this was a retrospective study of the patients’ chart records. All procedures were performed in accordance with the principles of the Declaration of Helsinki.

**FIGURE 2:**
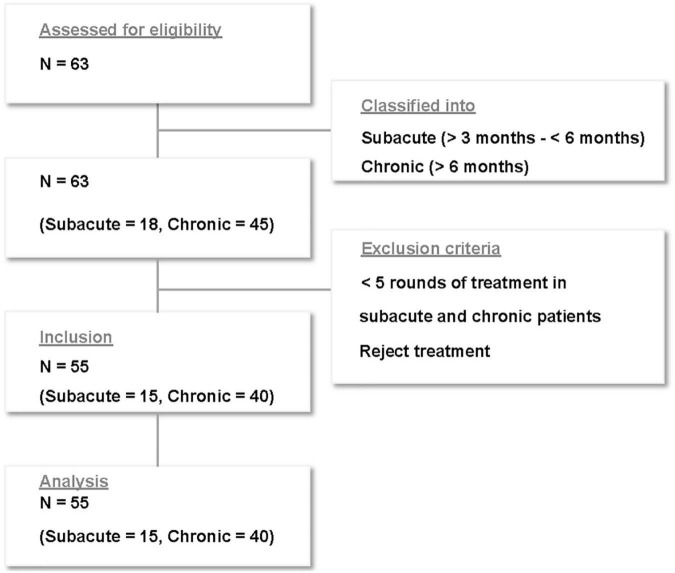
Flow chart of patient enrollment.

Of the 63 patients who received treatment for subacute and chronic tinnitus at Sirh’s Private Pain Clinic in Seoul between January 2, 2017, and December 30, 2018, a total of 55 patients (subacute, *n* = 15; chronic, *n* = 40) met the inclusion criteria and were included in this study (Figure 2).

### Inclusion and Exclusion Criteria

The inclusion criteria were as follows:

1. a self-reported T-VAS score of ≥5 for subacute and chronic tinnitus,
2. ≥5 rounds of integrative treatment,
3. intractable or recurrent tinnitus with a duration >3 months for subacute tinnitus and >6 months for chronic tinnitus,
4. absence of substantial benefits or patient satisfaction after medication and other treatments,
5. subacute and chronic tinnitus unilateral or bilateral, and
6. subjective tinnitus regardless of whether accompanied by sensorineural hearing loss, objective or somatic tinnitus, or unidentified brain diseases.

The exclusion criteria were as follows:

1. a pre-treatment T-VAS score <5 for subacute or chronic tinnitus,
2. <5 rounds of our integrative treatment,
3. patient rejection of our treatment method,
4. acute tinnitus (<3 months), or
5. purely objective or somatic tinnitus.

All included patients had previously received verum acupuncture with reinforcing-reducing manipulation, and complementary or medical therapies including intratympanic steroid injections, repeated conventional nerve blocks (Tables 1, 2).

**TABLE 1:** Patients with subacute tinnitus: demographic data, visual analog scale (VAS) scores, and clinical findings.

	Tinnitus-VAS (T-VAS) scores						
Age/sex, duration (months)/etiology	Before Tx	During Tx	At the end of Tx	One year follow up	Number of procedures	Previous Tx history	Location, sound types, and physical findings
48/F, 3 idiopathic	10	2	0	0	11	Intratympanic steroid injection 4 ×	Both sides, escaping steam (hissing)
40/M, 5 neck	6	1	0	0	5	acupuncture, medication	Lt., escaping steam (hissing), buzzing, cervical HNP, aggravation on neck motion
27/F, 3–4 idiopathic	8	3	0	0	15	medication	Rt. > Lt., ringing, escaping steam (hissing)
67/M, 6 idiopathic	6	3	1	1	13	medication	Both, buzzing, ear fullness
59/M, 6 idiopathic	8	4	3	4	15	medication	Both, (Lt. > Rt.) crickets, heavy drinker, smoker (2 packs/day)
62/M, 4 TMJ	7	3	1	1	10	Intratympanic steroid injection 8 ×	Rt., escaping steam (hissing), wind buzzing, moderate deafness, aggravation on palpation of the TMJ
58/M, 3 neck	7	3	0	0	10	medication	Rt., escaping steam (hissing), wind aggravation on neck motion
50/F, 4 trauma	7	3	1	1	9	medication	Both (Lt. > Rt.), buzzing, developed following traffic accident, discomfort with neck flexion
34/M, 3 idiopathic	7	1	0	0	6	medication	Lt., escaping steam (hissing), typewriter
29/M, 4 neck	8	2	1	1	6	medication	Rt., escaping steam (hissing), typewriter, buzzing, aggravation on neck motion
57/F, 3 idiopathic	7	1	0	0	7	medication	Lt.
69/M, 4 idiopathic	5	3	1	1	8	medication	Lt., crickets
40/M, 4 idiopathic	6	2	0	0	13	Intratympanic steroid injections 3×, acupuncture, herbal medicine	Rt., buzzing
52/M, 4 idiopathic	8	3	0	0	14	medication	Lt., escaping steam (hissing)
66/M, 4 idiopathic	7	2	1	1	5	Medication, dietary supplement, acupuncture	Both (Lt > Rt.), ringing
Means	7.13	2.40	0.60	0.67	9.8		

*M, male; F, female; T-VAS, tinnitus visual analog scale; MRI, magnetic resonance imaging; Lt., left; Rt., right; Tx, treatment; T-VAS during treatment, T-VAS at session 4; HNP, herniation of the nucleus pulposus; TMJ, temporomandibular joint Idiopathic, 10; head-and-neck-associated, 3; trauma, 1; TMJ disorder, 1 (including idiopathic hearing impairment, 1).*

**TABLE 2:** Demographic data, visual analog scale (VAS) scores and clinical data in chronic patients.

	Tinnitus-VAS (T-VAS) scores						
Age/sex, duration (y) etiology	Before Tx	During Tx	End of Tx	One year follow up	Number of procedures	Previous Tx history	Location, sound type and physical findings
44/F, 3 idiopathic	7	3	1	0	7	Medication, sound masking device	Both, cicadas
55/F, 6 neck	7	1	0	0	5	Medication	Lt., escaping steam (hissing) vertigo, posterior neck pain especially with neck extension
55/M, 1 idiopathic	8	3	0	0	15	Intratympanic steroid injection 6 ×	Both (Lt. > Rt.), escaping steam (hissing) hearing impairment deafness (80 dB, 50 dB); after Tx, mild improvement
45/M, 3 idiopathic	6	2	0	0	12	Medication	Both, crickets Both: moderate deafness (50–60 dB)
72/F, 1 idiopathic	6	2	0	0	11	Medication	Both, ocean waves, pulsating
51/M, 25 idiopathic	6	2	1	0	6	various conservative Tx	Lt. > Rt., buzzing, escaping steam (hissing)
44/M, 2 idiopathic	8	2	2	2	5	Acupuncture, medication	Lt. > Rt., buzzing
28/M, 2 idiopathic	10	3	0	0	18	Acupuncture, medication	Lt. > Rt., combined sounds
56/M, 1 idiopathic	7	3	2	2	11	Medication, dietary supplements	Lt., buzzing with pulsating
62/M, 1 idiopathic	8	2	1	1	6	Acupuncture, medication, dietary supplements	Lt., escaping steam (hissing)
52/M, 1.5 idiopathic	7	4	2	2	8	Medication, dietary supplements	Lt., grinding steel
84/F, 5 presbycusis	10	7	5	6	11	Medication	Both, escaping steam (hissing) dizziness, hearing aid Lt. ear, total deafness (hearing aids)
70/F, 1 idiopathic	7	3	2	2	6	Medication, dietary supplements, acupuncture	Lt. > Rt., crickets
40/F, 1.5–2 idiopathic	8	3	0	0	13	Medication, dietary supplements	Both, grinding steel, pulsating
49/M, 1 neck	8	3	2	2	10	Medication	Both, (Lt. > Rt.), wind occurred after neck sprain aggravation on neck motion
59/M, 1 trauma	7	5	3	3	15	Intratympanic steroid injection 5×, medication, dietary supplements	Lt., cicadas, sirens occurred after bicycle accident
60/M, 3 idiopathic	8	5	4	3	6	Medication, dietary supplements, acupuncture, moxibustion	Both, buzzing
54/M, 4 idiopathic	8	3	0	0	15	Medication, dietary supplements, acupuncture, sound therapy, TMJ splint	Rt. > Lt., buzzing moderate deafness, hearing aid for 2.5 y
50/F, 1 neck	6	4	2	2	8	Tympanoplasty, medication, dietary supplements	Rt. > Lt., cicadas, grinding steel neck and thoracic pain
45/F, 5 idiopathic	8	3	1	0	10	Medication, dietary supplements	Both, cicadas moderate deafness, hyperacusis, dizziness
56/M, 2 idiopathic	8	4	2	2	7	Medication	Rt. > Lt., escaping steam (hissing) moderate deafness
39/F, 2 idiopathic	8	2	0	0	10	Medication, dietary supplements	Both
67/F, 1.5–2 idiopathic	10	4	3	3	8	Tympanoplasty, medication, dietary supplements	Both, grinding steel
39/M, 18 idiopathic	10	4	2	2	8	Medication (opioids)	Both, escaping steam (hissing) moderate deafness, earache
59/M, 7 idiopathic	6	3	1	1	8	Medication, dietary supplements	Lt. > Rt., crickets
46/F, 10 idiopathic	10	5	2	2	18	Medication, dietary supplements	Lt. > Rt., escaping steam (hissing) dizziness
49/F, 1 idiopathic	8	3	2	2	18	Medication (Rivotril^®^, anticonvulsant)	Lt. > Rt.
47/M, 1 idiopathic	6	2	1	1	5	Medication, dietary supplements	Rt. > Lt., escaping steam (hissing)
45/M, 2 idiopathic	6	1	0	0	6	Medication	Lt., escaping steam (hissing) operation
65/F, 2 idiopathic	7	2	0	0	10	Medication, dietary supplements	Both, cicadas
52/M, 13 ototoxicity	10	6	4	5	15	Various Tx	Rt., cicadas occurred after chemotherapy (lymphoma)
57/F, 8 idiopathic	8	8	8	8	7	Intratympanic steroid injection 4×	Both, Rt. > Lt., wind escaping steam (hissing)
75/F, 6 idiopathic	10	3	1	1	8	Acupuncture, medication,	Lt., escaping steam (hissing) hearing aids, insomnia
27/M, Rt.: 2; Lt.: 3 idiopathic	6	2	0	0	11	intratympanic steroid injection > 10×	Both, Lt. > Rt., deafness (40–60 dB↑), earfullness
23/F, 1 TMJ	6	2	0	0	10	Medication	Both, Lt. > Rt., buzzing, escaping steam (hissing) TMJ treatment. + TMJ pain/instability, facial pain
31/M, 1 idiopathic	7	4	2	3	8	Intratympanic steroid injection 4×, intratympanic stem cell injection test	Both, Lt. > Rt., crickets both mild deafness (30–40 dB)
57/F, 3 idiopathic	8	1	0	0	7	Various conservative Tx	Lt. > Rt., buzzing occurred after otitis media, earfullness
67/M, A few decades TMJ	10	6	3	4	12	Acupuncture, moxibustion, medication	Both, buzzing both, severe deafness (90–100 dB), aggravation on head, neck, and TMJ motion
58/F, 5 idiopathic	8	1	0	0	7	Acupuncture, medication	Lt., crickets
70/F, 10 idiopathic	7	4	2	2	10	Acupuncture, medication	Lt., escaping steam (hissing)
Means	7.73	3.25	1.53	1.53	9.775		

*M, male; F, female; T-VAS scores, tinnitus visual analog scale scores; MRI, magnetic resonance imaging; Lt., left; Rt., right; Tx, treatment; T-VAS during treatment, at session 4; TMJ, temporomandibular joint. Idiopathic, 32; head-and-neck-associated, 3; trauma, 1; TMJ disorder, 2; ototoxicity, 1; idiopathic hearing impairment, 11 (including presbycusis, 1).*

We excluded patients with tinnitus with a T-VAS score <5 because they did not usually seriously complain of tinnitus loudness, emotional stress, or impairment of quality of life, had rarely visited the clinic and either had specific medication preferences or rejected our treatment for financial reasons even if they visited the clinic.

### T-VAS Score as a Measure to Evaluate Treatment Effect

There is no consensus regarding the best questionnaire to use to assess tinnitus loudness, distress, quality of life, and treatment effect, simply and simultaneously.

Currently, the VAS score is one of the most used measures for assessing tinnitus loudness, annoyance, distress, and effect on quality of life, as well as pain intensity, and has shown adequate validity and reliability in several studies (Figueiredo et al., 2009; Adamchic et al., 2012; Raj-Koziak et al., 2018; Kojima et al., 2019).

Therefore, HM Sirh devised and applied a simplified pictorial smiley score for tinnitus (T-VAS). It is easy to understand at first glance and is a modification of the conventional VAS, assigning levels ranging from 0 to 10: 0, no tinnitus and 10, the loudest tinnitus imaginable (Figure 1). This scale simply, quickly, and simultaneously assesses loudness, distress, and quality of life in patients with tinnitus and is a more convenient alternative to the Tinnitus Handicap Inventory (THI) (Newman et al., 1996).

Prior to the patients’ rating, patients were instructed about pictorial T-VAS score by doctors and comprehensibly understood its meaning, combining mainly loudness with distress and quality of life. Then, the patients reported a T-VAS score as a single score before each intervention at pre-treatment, and at the 4*^th^* and last treatment session. The rating itself was done by the patients alone without interference or help from doctors.

### Classification of Tinnitus According to Duration

Tinnitus can be classified as acute, subacute, or chronic, depending on the duration of the symptoms. However, there are no clearly defined criteria for classifying tinnitus based on duration after the first onset.

Therefore, in the present study, acute, subacute, and chronic tinnitus were defined as a condition lasting <3 months, 3–6months, and >6 months, respectively, as duration is important for selecting tinnitus treatment modalities.

### Procedure

Performing electrical or manual stimulation near large nerves can be risky. Currently, there are no reported literature on the effects and methods of innocuous mechanical nerve stimulation through needle placement near large nerves without electrical or manual stimulation. However, in the last 15 years, we have investigated and observed that the facial nerve and assumed vestibulocochlear nerve and its pathways can be stimulated through placement of a thin injection needle with a hubcap close to the facial nerve, without electrical and manual stimulation, to avoid the known risks and to improve treatment effects.

Therefore, in our integrative treatment method, combined simultaneous bilateral nerve blocks were administered to the facial nerve (7th cranial nerve, CN VII) and auriculotemporal nerve (a branch of the trigeminal nerve—CN V) after inducing innocuous, mechanical nerve stimulation using a thin injection needle via the CN V, CN VII, and the assumed vestibulocochlear (8th cranial nerve, CN VIII) pathways.

In combined tinnitus, we have occasionally also performed maxillary and mandibular nerve blocks after stimulating the maxillary and mandibular nerves through the infrazygomatic arch approach, along with TPI and prolotherapy in the upper cervical areas (above C3 vertebra) in patients with myofascial problems such as masseter, medial, and lateral pterygoid muscles spasm/contracture or injuries to the TMJ, upper cervical muscles, tendons, or ligaments.

We did not perform conventional nerve blocks as a control due to ethical considerations, poor efficacy of the nerve blocks since almost none of our patients with subacute or chronic tinnitus could be treated by conventional nerve blocks in light of literature guidelines (Tunkel et al., 2014; Cima et al., 2019; Ogawa et al., 2020) and our experience.

Instead, we compared the effects of our treatment method between patients with subacute and chronic tinnitus, between pre-treatment and mid-treatment timepoints, between pre-treatment and end of treatment, and between mid-treatment and end of treatment.

All patients were followed-up for approximately 1 year, including the treatment period, according to our clinical policy. The treatment outcomes were evaluated using the T-VAS score reported by patients before intervention at pre-treatment, each treatment visit, and at the end of the treatment, through chart review and by telephone interview after the end of the treatment.

### Newly Designed Hypodermic Needle

**FIGURE 3:**
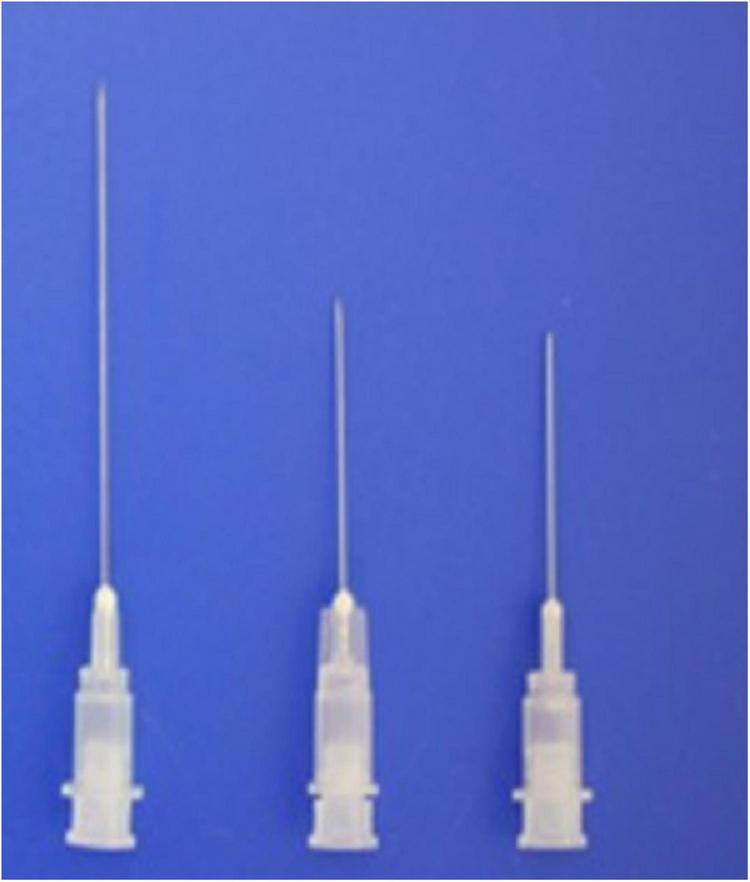
Shown are the novel 1. 9-, 2. 2-, and 3.8-cm-long, thin, 30-G hypodermic needles with needle hub caps used in the present procedures.

Newly designed 30G, 1.9– 3.8-cm thin hypodermic needles (Figure 3) were used for treating chronic intractable tinnitus, diseases, and pain refractory to medical, neurostimulation, neuromodulation, and surgical treatments. The new needles prevented complications such as unintentional nerve and vessel injuries by the needles or by anesthetic injection, by having a transparent plastic needle hub and hub cap that could be used to confirm the presence or absence of blood or cerebrospinal fluid without the need for aspiration.

Through this needle, we could perform the repeated nerve blocks more effectively by opening up the hubcap for repeated nerve stimulations in the same targeted site at each intervention.

A patent for the new needle (injection needle with a plastic needle hub cap) has been registered with the Korean Intellectual Property Office; inventor, Hun Man Sirh.

### Facial Nerve Approach

**FIGURE 4:**
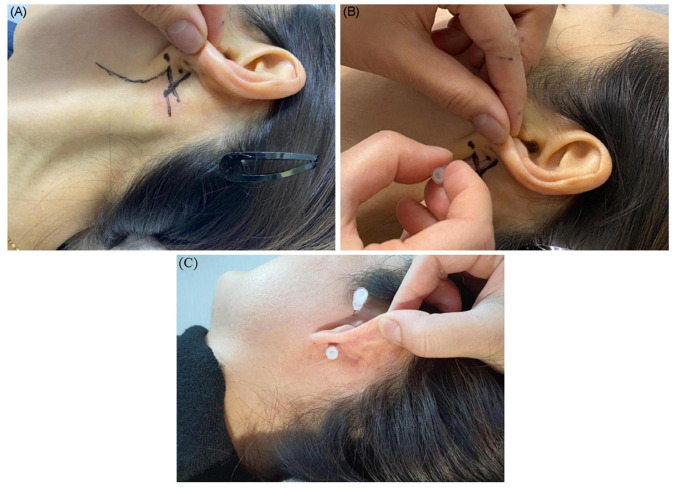
Shown is the needle entry point for administering a facial nerve block. **(A)** Entry point; **(B)** needling direction. **(C)** Needle placement for the facial nerve and auriculotemporal nerve stimulation and block.

The entry site was the area where the skin is indented on finger palpation (usually using the fifth fingertip) between the posterior border of the mandibular ramus and the anterior border of the mastoid process, above the lowest point of the earlobe (Figures 4A,B).

**FIGURE 5:**
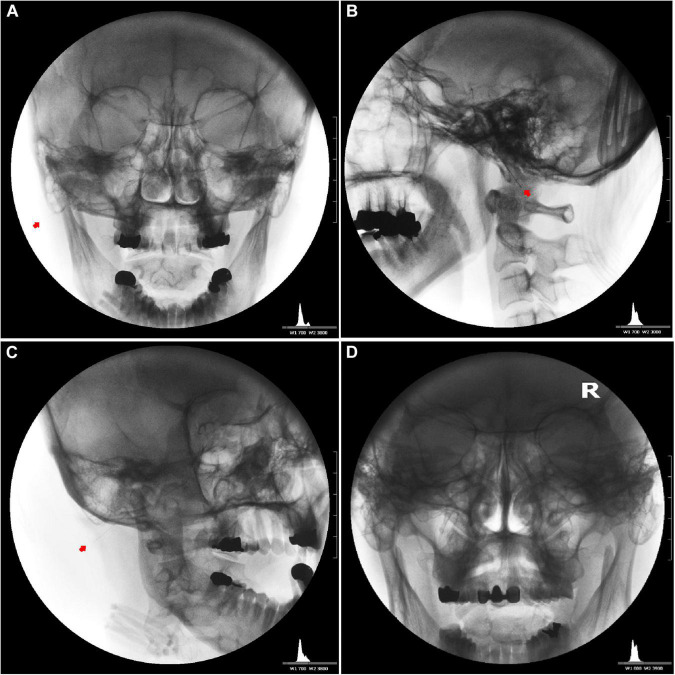
Needle placement for the induction of bilateral facial nerve block after inducing vestibulocochlear (8th cranial nerve, CN VIII) stimulation indirectly via the facial nerve. Unilateral anteroposterior **(A)**, lateral **(B)**, oblique **(C)**, and bilateral anteroposterior **(D)** fluoroscopic views showing the tips of the needles close to the stylomastoid foramen between the mastoid process and the styloid process.

We inserted the needle slightly superiorly to a depth of 1.9–2.5 cm at the entry site with the patients’ mouth open and head turned 30–45° to the opposite side (Figures 5A,C,D). Then, we placed the needle near the facial nerve (CN VII) around the expected stylomastoid foramen (usually 19–25 mm deep from the entry point) with the mouth closed for 20–40 min (Figure 5B).

The needle was inserted to a depth of 1.9–2.5 cm according to the muscle thickness between the posterior border of the mandibular ramus and the anterior border of the mastoid process. This careful needle insertion would avoid facial nerve injury and long-term facial nerve palsy. In addition, careful needle insertion kept the patients comfortable during needle placement and avoided needle grasp by muscle spasm.

### Auriculotemporal Nerve Approach

We inserted and placed the needle perpendicular to the skin to a depth of 0.5–1 cm near the auriculotemporal nerve, avoiding the adjacent superficial temporal artery through the vertical cleft just 0.5–1 cm anterior to the tragus of the ear with the patient’s mouth open and eyes facing front (Figure 4C). We could then observe needle pulsation due to the adjacent superficial temporal artery.

### Integrative Treatment Method and Course

We then stimulated the facial and auriculotemporal nerves by placing the needles innocuously without manual or electrical stimulation for 20–40 min (mean placement duration, 20–30 min in the subacute group; 30–40 min in the chronic group) to increase the treatment effect by nerve stimulation because to a considerable degree, the magnitude and duration of tinnitus relief varies according to needle placement time.

The procedure usually takes 50–60 min in total. None of the patients complained of any discomfort, except for mild needle insertion pain or postintervention pain for 1–2 days.

In unilateral tinnitus, either <0.5 mL of 0.5% lidocaine was administered, or needle stimulation alone was performed on the normal ear side, and 1 mL of 0.5% lidocaine was injected after hypodermic needle stimulation on the affected side. For tinnitus occurring on both sides, 1 mL of 0.5% lidocaine was injected on both sides of the facial nerve. In addition, 0.5 mL of 0.5% lidocaine was injected near the auriculotemporal nerve on the affected side after needle stimulation.

Transient facial palsy (5–15 min) after nerve block was a good sign for tinnitus treatment because it confirmed that the needle was close to the facial nerve.

We performed the procedure 2–3 times a week for the first 2–3 weeks. After confirming a marked continuous decrease or disappearance of tinnitus, we tapered the treatment to 1 or 2 times per week and then once every 1 or 2 weeks.

Treatment was terminated when the total number of procedures performed was more than 10, when patients felt comfortable for over 2 weeks during the treatment period, or when patients wanted to stop the treatment.

### Statistical Analysis

To evaluate the treatment effects in the patients with subacute and chronic tinnitus and to identify the difference in effect between these patients based on tinnitus duration, we compared the changes in the T-VAS scores between pre-treatment and during treatment (at the 4th treatment session), pre-treatment and at the end of the treatment, and during treatment and at the end of the treatment. We also compared the treatment effect between the subacute and chronic groups at the end of the treatment.

**FIGURE 6:**
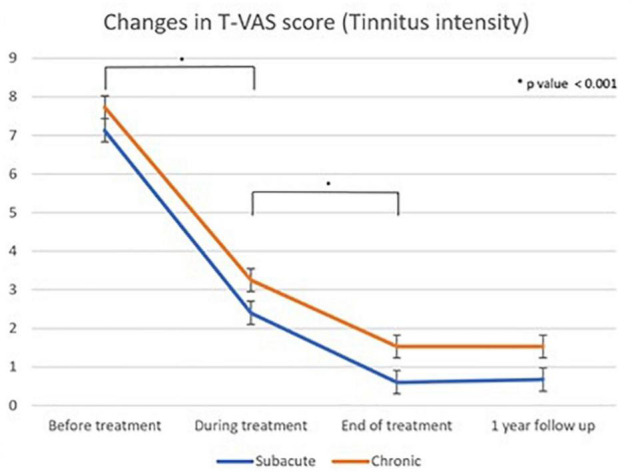
Linear graph of the Dunnet T3 *post hoc* test of changes in T-VAS score (tinnitus intensity) as measured using the Tinnitus visual analog scale score between different treatment time points: subacute and chronic tinnitus group “During treatment” corresponds to 4th treatment session, “end of treatment” refers to cessation of all treatments, and “one year follow up” refers to 1 year after the first treatment.

Statistical analyses were performed using SPSS (version 18.0, SPSS Inc., Chicago, IL, United States). A one-way repeated measures ANOVA and Dunnett’s T3 *post hoc* test were performed to assess the significant differences in the T-VAS scores between the two groups (Tables 3, 4 and Figure 6).

**TABLE 3:** Results of the Dunnet T3 *post hoc* test regarding changes in the T-VAS score (tinnitus intensity) between different treatment time points: subacute tinnitus group.

Group (a) VAS score (preceding T-VAS)	Group (b) VAS score (subsequent T-VAS)	Mean difference (a - b)	Standard deviation	Standard error	*p*-value	95% CI
Before treatment (mean T-VAS, 7.13)	During treatment (mean T-VAS, 2.40)	4.733	0.386	0.386	<0.001*	(3.75, 5.72)
	At the end of treatment (mean T-VAS, 0.60)	6.533	0.374	0.374	<0.001*	(5.58, 7.49)
	One year follow up (mean T-VAS, 0.67)	6.467	1.55	0.401	<0.001*	(5.61,7.33)
During treatment (mean T-VAS, 2.40)	At the end of treatment (mean T-VAS, 0.60)	1.800	0.318	0.318	<0.001*	(0.99, 2.61)
	One year follow up (mean T-VAS, 0.67)	1.733	0.884	0.228	<0.001*	(1.24,2.22)

T-VAS, tinnitus visual analog scale; CI, confidence interval; *, significant difference. “During treatment” corresponds to 4th treatment session, and “end of treatment” refers to cessation of all treatments.

**TABLE 4:** Results of the Dunnett T3 *post hoc* test regarding changes in the T-VAS score (tinnitus intensity) between different treatment time points: chronic tinnitus group.

Group (a) VAS score (preceding T-VAS)	Group (b) VAS score (subsequent T-VAS)	Mean difference (a - b)	Standard deviation	Standard error	*p* value	95% CI
Before treatment (mean T-VAS, 7.73)	During treatment (mean T-VAS, 3.25)	4.475	0.335	0.335	<0.001*	(3.66, 5.29)
	End of treatment (mean T-VAS, 1.53)	6.200	0.343	0.343	<0.001*	(5.36, 7.04)
	One year follow up (mean T-VAS, 1.53)	6.200	1.78	0.282	<0.001*	(5.63,6.77)
During treatment (mean T-VAS, 3.25)	End of treatment (mean T-VAS, 1.53)	1.725	0.368	0.368	<0.001*	(0.83, 2.62)
	One year follow up (mean T-VAS, 1.53)	6.200	0.816	0.129	<0.001*	(1.46,1.99)

T-VAS, tinnitus visual analog scale; CI, confidence interval; *, significant difference “During treatment” refers to 4^th^ treatment session, and “end of treatment” refers to cessation of all treatments.

A two-way repeated measures ANOVA was performed to compare the differences in the treatment effect between the subacute (mean T-VAS, 0.60) and chronic groups (mean T-VAS, 1.52) at the end of the treatment. Statistical significance was set at *p*-value < 0.05 (Tables 3, 4).

## Results

### Demographic Characteristics

The demographics, T-VAS scores, and previous treatments of the patients with subacute and chronic tinnitus are summarized in Tables 1, 2. The study participants included 15 subacute [11 men (73%), mean age 50.5 years, SD 13.81 years, min 27 years, max 69 years] and 40 chronic patients [21 men (52.5%), mean age 52.6 years, SD 13.33 years, min 23 years, max 84 years].

Tinnitus duration varied from 4 to 6 months in subacute patients and over 6 months to a few decades in chronic patients.

The etiologies for subacute tinnitus included: idiopathic, 10; head-and-neck-associated, 3; trauma, 1; and TMJ disorder, 1 (including idiopathic hearing impairment, 1). The etiologies for chronic tinnitus included: idiopathic, 32 (hearing impairment, 11, including presbycusis, 1); head-and-neck-associated, 3; trauma, 1; TMJ, 2; and ototoxicity, 1. The details are summarized in Tables 1, 2.

Some patients [3/15 (20%) in subacute and 5/40 (12.5%) in chronic tinnitus] had neck and TMJ problems on physical examination (Tables 1, 2). An average of approximately 10 procedures [subacute: mean (range) = 9.8 (5–15); chronic: 9.77 (5–18)] were performed.

### Treatment Effects

A few patients [1/15 (about 6.7%) in subacute, 5/40 (12.5%) in chronic] reported unsatisfactory results (<70% improvement) as follows: T-VAS score reduced from 8 to 3 in one subacute patient, from 10 to 5, 4, and 3 in three chronic patients, respectively, and from 8 to 8 and 4 in two patients, respectively (Tables 1, 2).

Tables 3, 4 show the results of Dunnett’s T3 *post hoc* test regarding changes in the T-VAS score between pre-treatment, mid-treatment, end of treatment, and 1 year follow up in subacute and chronic tinnitus. A significant probability of 0.001 was demonstrated for improvement in tinnitus during and after treatment compared to pre-treatment. When the 4th session (“mid-treatment”) was compared to pre-treatment, the mean T-VAS score was reduced from 7.13 to 2.4 in the subacute tinnitus and from 7.73 to 3.25 in the chronic group. In contrast, for the comparison between pre-treatment and after treatment, the score was reduced from 7.13 to 0.6 in the subacute group and from 7.73 to 1.53 in the chronic group (*p* < 0.001) (Tables 3, 4).

Additionally, within four treatment sessions, most patients [14/15 (93%) in subacute and 28/40 (70%) in chronic tinnitus] reported a transient reduction or fluctuation of tinnitus loudness.

A two-way repeated measures ANOVA showed that the difference in treatment effect between the subacute (mean T-VAS, 0.60) and chronic groups (mean T-VAS, 1.52) at the end of treatment was statistically insignificant (*p* = 0.871). The treatment effect in both groups was excellent.

The results of multiple regression analyses showed that the treatment effect may also vary by age (*p* = 0.001) and the number of treatment procedures (*p* = 0.017).

Tables 5, 6 show the VAS outcomes in the excluded eight patients (chronic, 5; subacute, 3). The results were very satisfactory with no reports of transient reduction or fluctuations in tinnitus loudness within four treatment sessions or tinnitus recurrence a few hours or few days later.

**TABLE 5:** Demographic data, visual analog scale (VAS) scores, and clinical data in excluded subacute tinnitus patients.

	Tinnitus-VAS (T-VAS) scores					
Age/Sex Duration (month)	Before Tx	During Tx	At the end of Tx	No. of procedures	Previous Tx history	Location, sound types, and physical findings
26/M 6	6–7	2	1–2	3	Medication	buzzing
36/M 6	6	2	1–2	3	Medication, acupuncture	escaping steam (hissing) Occurred after cervical extracorporeal shock wave therapy
43/M 3–4	6–7	0	0	3	Medication, acupuncture	buzzing

**TABLE 6:** Demographic data, visual analog scale (VAS) scores, and clinical data in excluded chronic tinnitus patients.

	Tinnitus-VAS (T-VAS) scores					
Age/Sex Duration (y)	Before Tx	During Tx	At the end of Tx	No. of procedures	Previous Tx history	Location, sound types, and physical findings
51/F 30	6	2	1–2	4	Acupuncture, medication	Lt. > Rt. buzzing
40/M 2	8	0	0	4	Medication, dietary supplement	Both Buzzing, pulsating
58/M 3	10	2–3	2–3	1	Medication, dietary supplement	Cicadas
53/M 30	10	3	2–3	2	Acupuncture, dietary supplement	Ringing
46/F 0.5–1	8	1–2	1–2	3	Acupuncture, Medication, dietary supplement	Cicadas

## Discussion

Our integrative treatment was designed to treat patients with subacute and chronic tinnitus refractory to conventional, medical, and other treatments, including neuromodulation and neurostimulation. At the end of the treatment, the mean T-VAS score was reduced significantly from 7.13 to 0.6 in the subacute group and from 7.73 to 1.53 in the chronic group, and we observed a statistically insignificant (*p* = 0.871) difference in the treatment effect between the subacute (mean T-VAS, 0.60) and chronic tinnitus groups (mean T-VAS, 1.52). Our results demonstrate that repeated administration of combined modified nerve blocks and auditory and non-auditory nerve stimulation using thin hypodermic needles could enhance the effects and duration of nerve blocks and stimulation-induced relief in tinnitus via the V, VII, and VIII cranial nerve pathways.

Several tinnitus therapies, such as sound therapy, pharmacotherapy, neuromodulation, neurostimulation, and cognitive behavioral therapy (CBT), are beneficial (De Ridder et al., 2014; Yuan et al., 2018; Lefaucheur et al., 2020; Ogawa et al., 2020). Of the above modalities, the tinnitus clinical guidelines strongly recommend only CBT (Tunkel et al., 2014; Cima et al., 2019; Ogawa et al., 2020).

Indeed, there is no one effective treatment modality for tinnitus (Cederroth et al., 2019; Mcferran et al., 2019) because any single modality, including CBT, individualized transcranial magnetic stimulation (Lefaucheur et al., 2020), electrical neurostimulation, and neuromodulation (De Ridder et al., 2014; Yuan et al., 2018; Deklerck et al., 2020; Kochilas et al., 2020) may be promising but does not treat the multifactorial causes and underlying neuropathophysiological mechanisms of tinnitus.

Recent studies have reported various bimodal neuromodulation and neurostimulation treatments, such as the combination of auditory and somatosensory electrical stimulation of the tongue (Conlon et al., 2019, 2020), cheek, or neck (Marks et al., 2018) and the combination of vagal and auditory stimulation (Kochilas et al., 2020) or transcutaneous vagal nerve stimulation paired with notched music (Shim et al., 2015). These interventions try to compensate for auditory deafferentation by stimulating the somatosensory system (Conlon et al., 2019, 2020; Langguth, 2020) or modulating abnormal activity in the central auditory pathways by reversing the pathological neural activity through inducing frequency-specific long-term depression (Marks et al., 2018; Langguth, 2020).

Although a large, randomized study reported persistent positive improvements for 12 months after bimodal neuromodulation (Conlon et al., 2020), these procedures are occasionally used as the treatment of last resort when all other treatment modalities have failed.

In addition, vagal nerve stimulation alone or auditory or somatosensory stimulation alone without vagal nerve stimulation have been shown to be ineffective in treating chronic tinnitus (Kreuzer et al., 2014; Shim et al., 2015; Marks et al., 2018; Langguth, 2020; Stegeman et al., 2021).

To the best of our knowledge, there is no scholarly evidence on the effects of vagal nerve block after innocuously stimulating the vagus nerve, or vagal nerve block alone for tinnitus. Previously, we have tried to perform vagal nerve block after innocuously stimulating the vagus nerve, running just under and medial to the styloid process, using a thin needle without manual or electrical stimulation; however, we did not obtain good results in chronic sensorineural tinnitus (unpublished results). Importantly, in chronic idiopathic tinnitus, except for somatosensory tinnitus, treatment results were unsatisfactory when we did not stimulate the facial (VII) nerve around the expected stylomastoid foramen, which can stimulate, modulate, and restore CN VIII function based on mechanisms that compensate for auditory deafferentation (Conlon et al., 2019, 2020; Langguth, 2020) and the modulation of activity in the central auditory pathway (Marks et al., 2018; Langguth, 2020).

Despite the enormous progress in chronic tinnitus treatment, as of 2021, there are no known methods that can effectively stimulate the vestibulocochlear nerve (CN VIII), which is very closely associated with tinnitus. Hence, we developed the method of repeated nerve block following facial nerve stimulation around the stylomastoid foramen to therapeutically modulate the vestibulocochlear nerve in the peripheral brain structure.

The facial nerve has two roots, motor and sensory (nervus intermedius), and is potentially important for tinnitus treatment because it runs very close to the vestibulocochlear nerve (CN VIII) from the brainstem to the internal auditory canal, in which the vestibular-facial and inferior vestibular-cochlear nerves are connected (Tian et al., 2008).

Therefore, combined facial (CN VII) nerve block and stimulation by an injection needle is probably one of the most important and safest treatment methods that can strongly and indirectly stimulate the vestibulocochlear nerve (CN VIII), which is related to the modulation of auditory corticopetal (afferent) and corticofugal (efferent) pathways that diverge and converge (Pickles, 2015) at the multilevels of the auditory system as well as non-auditory brain areas, including intercollicular commissural connections (Malmierca et al., 2005; Orton and Rees, 2014), crossed connections (Glendenning et al., 1985; Orton and Rees, 2014), and each inferior colliculus, which mainly receive inputs from the opposite ear (Orton and Rees, 2014).

The cochlear nucleus is the first site of multisensory integration in the afferent auditory system. The dorsal cochlear nucleus integrates auditory input coming directly from the VIII*^th^* cranial nerve and somatosensory input coming indirectly from the Vth cranial nerve (Wu et al., 2015; Shore et al., 2016). Therefore, our method is assumed to stimulate the cochlear nucleus via trigeminal somatosensory input by combining auriculotemporal nerve and/or upper cervical nerve stimulation with a nerve block.

Hamani et al. (2006) observed a significant reduction in pain scores following the insertion of an electrode implanted in the thalamus and/or periaqueductal gray/periventricular gray (PAG/PVG) in the absence of electrical stimulation, which was associated with better treatment outcomes. Similarly, we frequently observed in patients with chronic intractable pain or trigeminal neuralgia that the magnitude of stimulation that was produced by simply placing thin hypodermic needles near the target nerves, without manual or electrical stimulation, correlated well with a successful outcome of our treatment, namely the analgesia and duration of pain relief achieved.

This study also showed that the magnitude and duration of tinnitus relief achieved by our treatment method, based on a similar mechanism, were often enough to produce successful outcomes.

Based on our findings, we hypothesized that our integrative treatment, combining repeated modified nerve blocks with facial and auriculotemporal nerve stimulation, can treat chronic intractable tinnitus through vestibular-facial and inferior vestibular-cochlear pathways, resulting in functional facial-cochlear stimulation, similar to the control of refractory pain via motor cortex and deep brain stimulation.

Some studies have reported somatosensory tinnitus due to myofascial etiologies, including the lateral pterygoid, masseteric, tensor tympani, stapedius, oral palate muscle, TMJ, and neck muscles (Jaeger and Maloney, 1999; Lockwood et al., 2002; Sanchez and Rocha, 2011). These muscles are innervated by the trigeminal, facial, glossopharyngeal (IX), vagus (X), and the first three cervical nerves (C1, C2, C3). Other studies have reported somatosensory tinnitus due to TMJ disorder and upper cervical ligament and tendon problems due to head and neck injuries or instability (Hackett et al., 2008; Levine and Oron, 2015; Shore et al., 2016; Michiels et al., 2018). Therefore, to treat somatosensory tinnitus due to muscle spasm and contracture, as well as tendon and ligament injuries in the middle ear, head, and neck region, we sometimes performed TPI, prolotherapy, and CN V, VII, IX, and X nerve blocks. Additionally, we administered bilateral treatment using commissural (Malmierca et al., 2005; Orton and Rees, 2014) and crossed connections (Glendenning et al., 1985; Orton and Rees, 2014) of auditory and non-auditory pathways, regardless of unilateral or bilateral tinnitus. This is because bilateral treatment is more effective than unilateral treatment. We also hypothesized that our integrative treatment can treat tinnitus by inducing adaptive (good) neuroplasticity, likely by modulating the bilateral auditory and non-auditory cortices via the central decussation and interconnection of the auditory and non-auditory tracts, managing either side of the lesion and directly treating the multiple causes of tinnitus.

In this study, we observed that patients experienced prolonged relief from tinnitus with increasing numbers of procedures. Moreover, when the tinnitus was treated or markedly reduced, distress levels went down and quality of life improved.

However, in several patients, failure to repeat the procedures triggered tinnitus recurrence a few hours or a few days later. Thus, for treating subacute and chronic intractable tinnitus, 10–15 procedures were needed to achieve long-term satisfactory tinnitus relief. Additionally, the treatment effect (change in T-VAS score) observed during the early treatment sessions correlated well with successful treatment outcomes. If the treatment effect was not good within four treatment rounds, a poor prognosis was usually observed. If tinnitus disappearance or significant tinnitus reduction was observed at the first treatment or within four treatment sessions, the treatment results were excellent. Therefore, tinnitus disappearance or degree of tinnitus relief should be evaluated during the first treatment or within four treatment sessions. However, the minimum required number of treatment sessions still needs to be determined.

Age and tinnitus duration were also associated with poor clinical outcomes because of the likelihood of chronic maladaptive neuroplasticity and complex causes (for example, age-related hearing loss, cochlear cell degeneration, or psycho-emotional pathology) of auditory and non-auditory systems. Therefore, tinnitus should be treated as early as possible after its onset because auditory maladaptive neuroplasticity refractory to the treatment occurs ≥3 months after tinnitus onset.

Our treatment method, without incurring electrical or mechanical injury to the cranial nerves V, VII, and VIII, strengthened the effects of therapeutic stimulation of the peripheral and central auditory nervous systems and nerve block in chronic and subacute tinnitus. These treatment outcomes suggest that extensive research on integrative needle treatment and electrical or magnetic neuromodulation and neurostimulation is needed.

Procedure-associated complications during tinnitus treatment should not be overlooked. In this study, to reduce the side effects of repeated use of a high concentration of lidocaine, we administered only 0.5% lidocaine during nerve blocks. Our integrative method is a notably safer, more effective, and minimally invasive treatment for tinnitus that had only negligible bleeding and bruising complications despite the use of anticoagulants, and no other major complications. Accordingly, our method may be considered as one of the primary treatment methods for tinnitus. Additionally, we have observed that typical responses associated with sensorineural tinnitus in the absence of repeated procedures are characterized by recurrence of tinnitus a few hours or a few days later and a transient reduction or fluctuation in tinnitus loudness within four treatment sessions.

We excluded eight patients due to the high possibility of somatic tinnitus and the high risk of statistical bias (Tables 5, 6). All patients paid for the treatment themselves, which was partially covered by health insurance.

Our study has several limitations. The 1-year follow-up in this study was relatively short. Therefore, long-term follow-up is required to investigate whether tinnitus relief is long-lasting. Moreover, we did not exclude all patients with brain lesions because some patients did not undergo magnetic resonance imaging for tinnitus. In addition, we did not differentiate between tinnitus with or without a combination of myofascial problems and/or injuries to the upper cervical muscles, tendons, and ligaments because we consider nerve block with facial nerve stimulation important and several patients had myofascial, tendon, and ligament problems. We did not perform validation tests such as the Tinnitus Handicap Inventory, Tinnitus Questionnaire, Tinnitus Functional Index, and Tinnitus Severity Index, which could have provided more detailed information.

We think that the T-VAS score will need to be further investigated for scholarly evidence, even if it is considered simple and convenient and is preferred by patients. Moreover, the possibility of placebo effect in our study is real. Therefore, randomized blinded controlled trials, without repeated nerve block after facial nerve stimulation or facial nerve stimulation, are needed to confirm and enhance the efficacy, potential, and benefits of our treatment method for tinnitus.

Notably, if our results can be confirmed in further large randomized controlled prospective studies, our integrative treatment will provide a new impetus to neuropathophysiological research on tinnitus and will be a promising treatment method for numerous patients with intractable tinnitus.

In conclusion, the duration of tinnitus relief or disappearance gradually increased in most patients as the total number of treatment procedures increased (minimum: 5, range: 5-15 in subacute, 5–18 in chronic). This study shows that our integrative treatment, combining repeated modified nerve blocks with stimulation of the peripheral and central auditory and non-auditory nervous systems via the V, VII, and VIII cranial nerve pathways, can be a promising intervention for patients with subacute or chronic tinnitus refractory to medical, conservative, and other treatment options. In addition, our treatment method will inform further research on the mechanisms of subacute and chronic tinnitus involving modulation of the ascending, interconnecting, crossing, and descending fibers of the auditory and non-auditory nervous systems and monitoring of the resulting neurotransmitter changes.

## Data Availability Statement

The raw data supporting the conclusions of this article will be made available by the authors, without undue reservation.

## Ethics Statement

The studies involving human participants were reviewed and approved by Wiltse Memorial Hospital Joint Research Ethics Committee in Suwon, South Korea (IRB approval number: 2021-W07). The ethics committee waived the requirement of written informed consent for participation. Written informed consent was obtained from the individual(s) for the publication of any potentially identifiable images or data included in this article.

## Author Contributions

HS and SJS were involved in study design, literature search, validation, data curation, and writing of the manuscript. SWS and HM organized the database, literature review, and prepared pictures and tables. HM checked the reproducibility of the results. All authors contributed to interpretation of the results, manuscript revision and reviewed the final version making the necessary changes, approved the submitted version, and agreed to be accountable for the content of the work.

## Conflict of Interest

The authors declare that the research was conducted in the absence of any commercial or financial relationships that could be construed as a potential conflict of interest.

## Publisher’s Note

All claims expressed in this article are solely those of the authors and do not necessarily represent those of their affiliated organizations, or those of the publisher, the editors and the reviewers. Any product that may be evaluated in this article, or claim that may be made by its manufacturer, is not guaranteed or endorsed by the publisher.

## Abbreviations

CBT: cognitive behavioral therapy
CN: cranial nerve
IRB: institutional review board
TMJ: temporomandibular joint
TPI: trigger point injection
T-VAS: tinnitus-visual analog scale.
